# Cause of death during 2009–2012, using a probabilistic model (InterVA-4): an experience from Ballabgarh Health and Demographic Surveillance System in India

**DOI:** 10.3402/gha.v7.25573

**Published:** 2014-10-29

**Authors:** Sanjay K. Rai, Shashi Kant, Puneet Misra, Rahul Srivastava, Chandrakant S. Pandav

**Affiliations:** 1 INDEPTH Network, Accra, Ghana; 2 All India Institute of Medical Sciences, New Delhi, India

**Keywords:** Ballabgarh, HDSS, cause of death, InterVA-4, mortality, rural India, verbal autopsy

## Abstract

**Objectives:**

The present study aimed to estimate the age and cause-specific mortality in Ballabgarh Health and Demographic Surveillance System (HDSS) site for the years 2009 to 2012, using a probabilistic model (InterVA-4).

**Methods:**

All Deaths in Ballabgarh HDSS from January 1, 2009, to December 31, 2012, were included in the study. InterVA-4 model (version 4.02) was used for assigning cause of death (COD). Data from the verbal autopsy (VA) tool were extracted and processed with the InterVA-4 model. Cause-specific mortality rate (CSMR) per 1,000 person-years was calculated.

**Results:**

A total of 2,459 deaths occurred in the HDSS during the year 2009 to 2012. Among them, 2,174 (88.4%) valid VA interviews were conducted. Crude death rate ranged from 7.1 (2009) to 6.4 (2012) per 1,000 population. The CSMR per 1,000 person-years over the years (2009–2012) for non-communicable diseases, communicable diseases, trauma, neoplasm, and maternal and neonatal diseases were 1.78, 1.68, 0.68, 0.49, and 0.48, respectively. The most common causes of death among children, adults, and the elderly were infectious diseases, trauma, and non-communicable diseases, respectively.

**Conclusions:**

Overall, non-communicable diseases constituted the largest proportion of mortality, whereas trauma was the most common COD among adults at Ballabgarh HDSS. Policy-makers ought to focus on prevention of premature CODs, especially prevention of infectious diseases in children, and intentional self-harm and road traffic accidents in the adult population.

Death recording by age, sex, and cause, and calculating mortality rates and differentials are central to evidence-based health policy, monitoring, and evaluation (1). Mortality levels are significant indicators of population health, and are of extreme importance to prioritize the goals of health systems and efficient resource allocation (2–4). About 46 million of the estimated 60 million deaths worldwide occur in developing countries (5). Unfortunately, up to 90% of them die at home. These deaths remain uncertified and unregistered due to weak civil registration system. Only 10 to 40% deaths occur in hospitals for which information on causes of death are available (6). India reported about 9 million deaths per year. Of these, more than three quarters occurred at home. Majority of these deaths did not have a medically certified cause of death (COD) (5).

Magnitude and trends in cause-specific mortality provide critical insights to both evolving and ignored diseases, and the efficiency of present disease control policy. Understanding of changes in leading causes of deaths is essential to modify the strategy for addressing current needs. It is widely recognized that where deaths are not routinely certified for their cause, verbal autopsy (VA) is the interim method of choice for documenting cause-specific mortality patterns (7).

Physician-assigned COD using VA has certain known limitations. Disagreement between the physicians (concordance between two physicians is required for assigning COD) lead to large proportion of causes of death being described as indeterminate (8, 9). Since the diagnostic criteria are not standardized for the physicians assigning COD, reproducibility of VAs over time, and in different settings, becomes one of the major limitations (9–11). Involvement of physicians is a costly and time-consuming process as they are already an overstretched resource in low-income countries (9, 12).

InterVA-4 model, a computer-based probabilistic model, can overcome these limitations, though it has its own limitations. Present study aimed to estimate the age and cause-specific mortality in Ballabgarh Health and Demographic Surveillance System (HDSS) site over the years 2009 to 2012 using a probabilistic model (InterVA-4).

## Methods

**Fig. 1:**
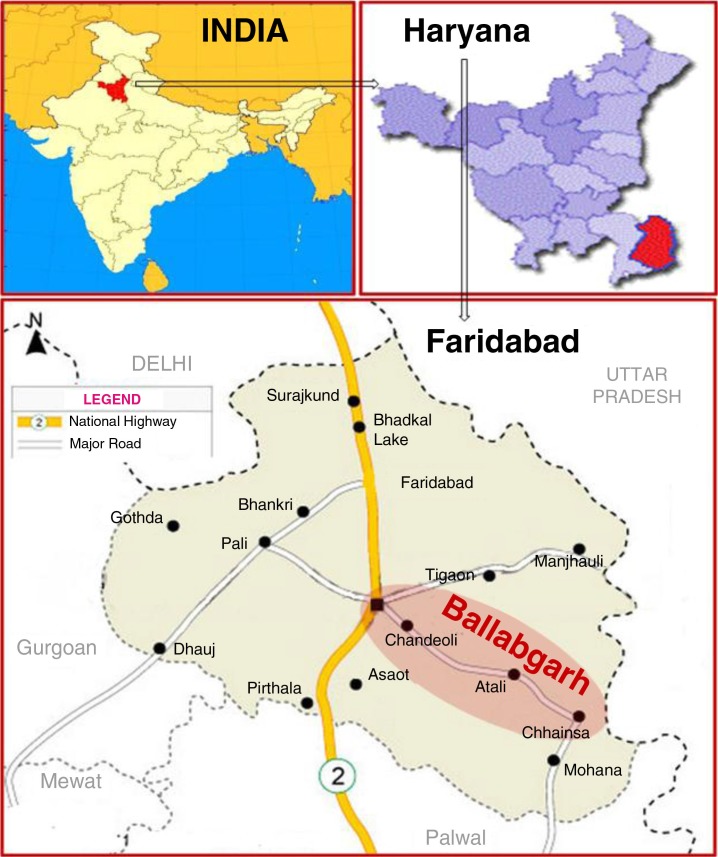
Map of Ballabgarh HDSS.

The Ballabgarh HDSS, also known as Comprehensive Rural Health Services Project, Ballabgarh, is situated in the northern part of India, and is around 22 miles from New Delhi (Fig. 1). At Ballabgarh HDSS, the population has been under surveillance since 1967 (13). The project is a collaboration between the All India Institute of Medical Sciences and the State Government of Haryana. The total population under surveillance was 92,070 (residing in 28 villages) as of December 31, 2012. The population was served by two Primary Health Center (PHCs) and 12 sub-center. The population of the villages ranged from 3,000 to 8,000. The villages were almost similar in terms of socio-economic status and cultural habits.

Multipurpose health workers visited all households covered under the HDSS twice a month, and provided basic health services such as immunization, family planning, and so on, along with the collection of information on vital events. VAs were conducted for all the deaths occurring in the HDSS area since 2002. Trained health workers administered the VA tool within 2 to 4 weeks of death. Physician-coded COD was assigned to all these VAs. All deaths that occurred between January 1, 2009, and December 31, 2012, were included in the study. Data were extracted for InterVA-4 application from the VA records.

### VA tool

The VA tool used in the present study was developed at Ballabgarh HDSS in 2002. This VA tool is based on the WHO VA tool but is short and takes less time to complete when compared with WHO VA tool. There were three types of VA tools for different age groups, namely 0–28 days, 29 days to 5 years, and more than 5 years. All the tools were validated, and had a good agreement with the WHO tool (14, 15). The VA tool contained a symptom checklist and a narrative portion.

### COD ascertainment: InterVA-4model

Data were extracted from the VA tool in accordance with the WHO 2012 VA standard, and processed with the InterVA-4 model (version 4.02), a probabilistic approach to COD determination (16). The data were entered into the already specified batchin.csv file format of InterVA-4, and a readable text output in log file format was chosen to assign the possible COD of each individual. Based on Bayes’ theorem (17), the InterVA-4 model calculates the probability of a set of causes of death given the presence of circumstances, signs, and symptoms (collectively called ‘indicators’) reported in VA interviews. The method is described in detail elsewhere (13, 18). Briefly, a finite number of COD are assigned to a predefined matrix of estimated probabilities of occurrence. The presence of indicators modifies the predefined probabilities of each COD upward or downward using Bayes’ theorem.

### Analysis

Causes of deaths were classified into six broad categories, namely non-communicable diseases, communicable diseases, trauma, indeterminate, neonatal/maternal, and neoplasm. These broad categories were further sub-classified. The classification was in-built in the InterVA-4 software and was based on ICD-10. Cause-specific mortality rate (CSMR) (standardized to INDEPTH standard population in terms of age group, sex, and year) was calculated over time. Person-years observed was calculated for all years and age group independently from the prospective database maintained at Ballabgarh HDSS. Person-years observed, and cause- and age-specific deaths were used to calculate CSMR, per 1,000 person-years. The age criteria selected for the present study were: children (0–14 years); adult (15–49 years); elderly (≥50 years). Yearly aggregated COD analysis was done so that the output could be compared with the mortality rates of other HDSSs using InterVA-4 model (19). All statistical analyses were performed with STATA release 11.1 software (Stata Corp., College Station, TX, USA).

## Results

A total of 2,459 deaths occurred in the HDSS during the period 2009 to 2012. Among them, 2,285 (93%) VAs were conducted. In the remaining 7% of the deaths, VAs could not be conducted mainly due to the unavailability of the informant, for example, family migrated after the death of the individual or refused to provide information needed for the VA tool. Among the VA interviews conducted, 2,174 (88.4%) forms were valid (completely filled) while the remaining 111 (4.5%) forms did not have sufficient information that was required for InterVA-4 to assign COD. The incomplete VA forms were rejected by the InterVA-4 during assignment of COD. The availability of adequately filled VA was 85.6, 87.5, and 89.9% among children (0–14 years), adults (15–49 years), and elderly (≥50 years), respectively.

**Fig. 2:**
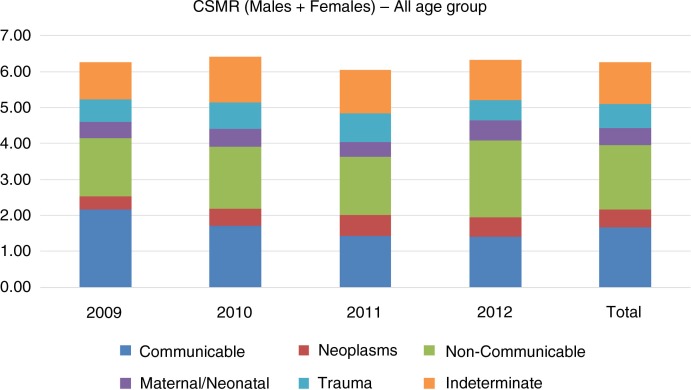
Cause-specific mortality rate (2009–2012) at Ballabgarh HDSS.

A declining trend of crude death rate (7.1 in 2009 to 6.4 in 2012 per 1,000 population) and infant mortality rate (53.7 in 2009 to 45.4 in 2012 per 1,000 live births) was observed in the Ballabgarh HDSS population (Table 1). A similar declining trend was also observed in child mortality rate (0 to 14 years) and adult mortality rate (15 to 49 years) whereas elderly mortality rate (>50 years) had a rising trend. The aggregated (2009–2012) CSMR in descending order is as follows: non-communicable diseases (1.78), communicable diseases (1.68), trauma (0.68), neoplasm (0.49), and maternal and neonatal diseases (0.48) (Fig. 2).

**Fig. 3:**
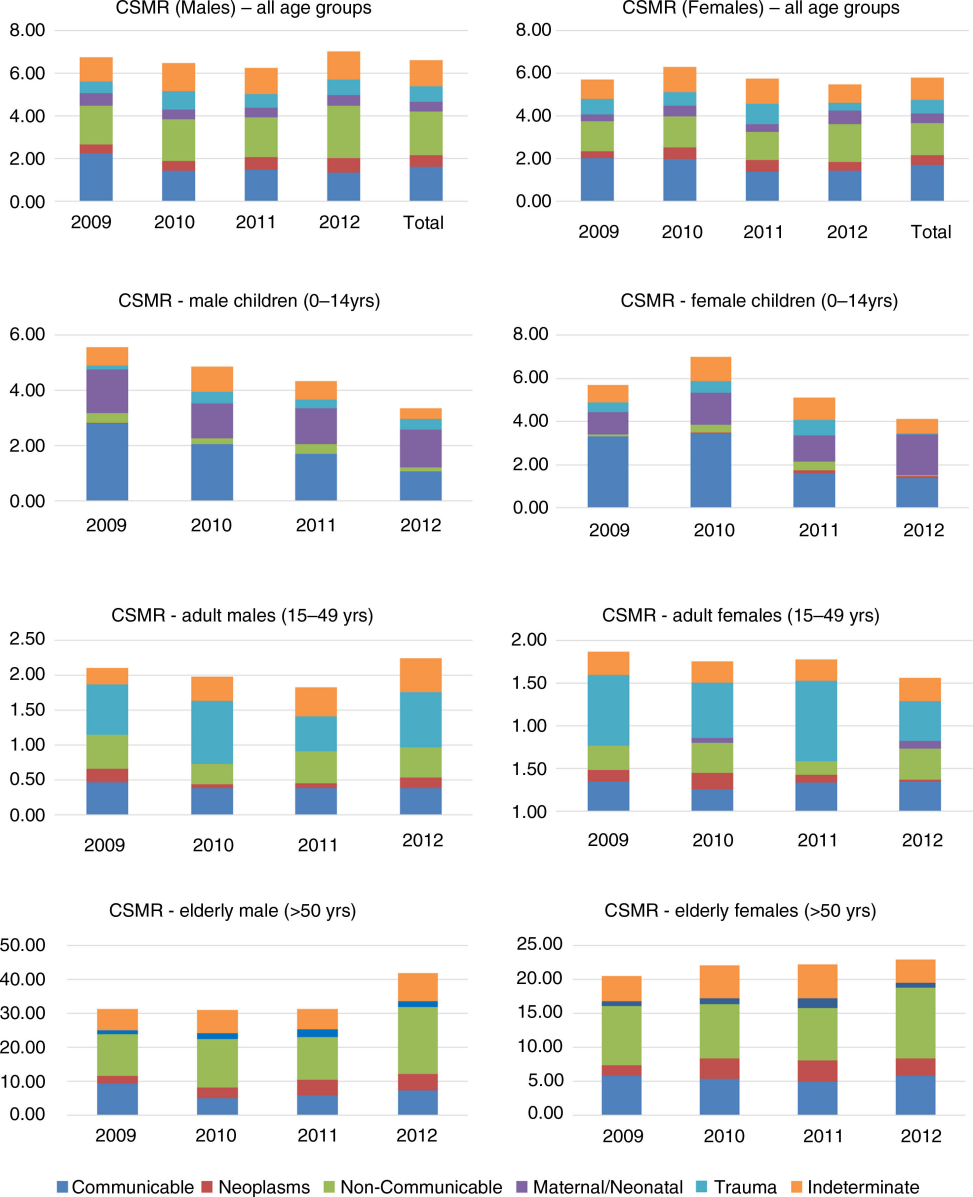
Cause-specific mortality rates (2009–2012) among males and females.

**Table 1:** Crude death rate and mortality trends at Ballabgarh HDSS, 2009–2012

					Mortality rate (deaths/1,000 person-years)		
					
Year	Person-years	Number of deaths	Crude death rate (per 1,000 population)	IMR^a^ (per 1,000 live births)	Children (0–14 years)	Adult (15–49 years)	Elderly (≥50 years)
2009	87,502	622	7.1	53.7	5.8	3.0	27.8
2010	88,830	635	7.0	56.1	5.5	2.6	29.9
2011	90,479	610	6.7	48.2	5.0	2.4	29.0
2012	90,976	592	6.4	45.4	3.8	2.4	31.2

a Infant mortality rate.

Over the years, CSMR (both male and female – all age group) for communicable diseases had declined (2.04 in 2009 to 1.45 in 2012) while that of non-communicable diseases had remained almost static. A declining trend was observed for communicable diseases among children (both male and female), and elderly male, whereas CSMR of communicable diseases fluctuated for adults (both male and female), and elderly females (Fig. 3). Among the elderly population, neoplasm was more common among males and also had an increasing trend. CSMR for neoplasm among elderly females was fluctuating. Trend of trauma was high among adult population but was fluctuating over the years for both males and females. Acute respiratory infection (ARI) (18.7%) was the commonest COD among infants and was followed by prematurity (18.0%). Among infants, Prematurity was more common among males (20.4%) when compared to females (15.1%). For children aged 1 to 4, diarrheal diseases was the commonest COD (22.5%) followed by ARI (19.2%) (Table 2). Trauma (37.3%) was the most common COD observed in the adult population followed by infectious diseases (18.9%) and non-communicable diseases (18.5%) (Table 3). Among trauma-associated deaths, the most common cause was transport accidents (13.3%), followed by intentional self-harm (12.4%). Intentional self-harm was slightly more common among females (13.6%) when compared to males (12.4%). Acute cardiac diseases and stroke were the most common causes among the non-communicable group. ‘Indeterminate’ was assigned as the COD in about 18% of all COD. It was highest for the elderly age group (19.8%) and lowest for children (15.0%).

**Table 2:** Cause of deaths in infant and 1 to 4 year-old children at Ballabgarh HDSS, 2009 to 2012

	Infants (%)			Children (1–4 years) (%)		
		
ICD-10 codes and cause of deaths	Male (109)	Female (86)	Total (195)	Male (85)	Female (80)	Total (165)
01.01 Sepsis (non-obstetric)	0.9	0.8	0.9	0.0	0.9	0.5
01.02 ARI, including pneumonia	18.9	18.4	18.7	11.2	27.4	19.2
01.03 HIV-/AIDS-related death	0.0	0.0	0.0	2.9	0.0	1.5
01.04 Diarrheal diseases	7.4	10.4	8.8	30.2	14.6	22.5
01.05 Malaria	1.3	1.7	1.5	18.3	18.6	18.4
01.07 Meningitis and encephalitis	1.2	2.2	1.6	1.8	0.0	0.9
01.10 Pertussis	0.2	0.0	0.1	0.0	1.9	0.9
01.99 Other and unspecified infect diseases	0.3	0.5	0.4	0.0	0.0	0.0
02.99 Other and unspecified neoplasms	0.0	0.0	0.0	0.9	0.0	0.4
03.01 Severe anemia	0.0	0.0	0.0	1.1	0.0	0.6
03.02 Severe malnutrition	0.9	0.7	0.8	0.6	1.8	1.2
03.03 Diabetes mellitus	0.0	0.3	0.1	1.9	0.0	1.0
04.99 Other and unspecified cardiac diseases	0.4	0.4	0.4	1.0	0.0	0.5
06.01 Acute abdomen	1.3	0.6	1.0	0.0	0.0	0.0
07.01 Renal failure	0.0	0.0	0.0	0.0	2.0	1.0
08.01 Epilepsy	1.0	0.9	1.0	1.8	0.0	0.9
10.01 Prematurity	20.4	15.1	18.0	0.0	0.0	0.0
10.02 Birth asphyxia	7.8	7.2	7.5	0.0	0.0	0.0
10.03 Neonatal pneumonia	10.9	9.0	10.0	0.0	0.0	0.0
10.04 Neonatal sepsis	4.0	5.8	4.9	0.0	0.0	0.0
10.06 Congenital malformation	1.7	1.9	1.8	2.0	0.0	1.0
10.99 Other and unspecified neonatal COD	6.2	7.1	6.6	0.0	0.0	0.0
12.01 Road traffic accident	0.0	0.0	0.0	2.0	4.1	3.0
12.03 Accidental fall	0.5	0.0	0.3	4.0	4.1	4.0
12.04 Accidental drowning and submersion	0.0	1.2	0.6	2.0	2.1	2.1
12.05 Accidental expos to smoke fire and flame	0.0	0.3	0.1	1.8	1.6	1.7
12.06 Contact with venomous plant/animal	0.3	0.0	0.2	0.0	2.1	1.0
12.99 Other and unspecified external COD	0.0	0.0	0.0	0.0	2.8	1.4
99 Indeterminate	14.2	15.5	14.8	16.3	16.0	16.1

**Table 3:** Cause of deaths at Ballabgarh HDSS by age and sex, 2009 to 2012

	Children (%): 0–14 years			Adult (%): 15–49 years			Elderly (%): ≥50 years		
			
Cause of death and ICD-10 codes	Male (267)	Female (233)	Total (500)	Male (271)	Female (172)	Total (443)	Male (728)	Female (503)	Total (1,213)
1-Infectious diseases	36.7	39.0	37.7	19.1	18.6	18.9	19.9	24.4	21.7
01.01 Sepsis (non-obstetric)	0.8	0.8	0.8	0.0	0.0	0.0	0.2	0.3	0.3
01.02 ARI, including pneumonia	16.0	18.7	17.3	0.7	1.1	0.9	5.1	5.4	5.2
01.04 Diarrheal diseases	11.4	10.4	11.0	0.7	1.0	0.8	1.1	1.7	1.4
01.07 Meningitis and encephalitis	1.2	2.1	1.6	0.8	0.8	0.8	0.0	0.0	0.0
Other and Unspecified	7.2	6.9	7.1	16.9	15.7	16.4	13.4	16.9	14.8
2-Neoplasms	0.2	1.0	0.6	5.8	6.3	6.0	11.7	12.2	11.9
02.01 Oral neoplasms	0.0	0.0	0.0	0.5	0.0	0.3	0.1	0.6	0.3
02.02 Digestive neoplasms	0.0	0.0	0.0	2.2	4.0	2.9	6.3	6.3	6.3
02.03 Respiratory neoplasms	0.0	0.0	0.0	0.4	0.1	0.3	1.8	2.0	1.9
02.04 Breast neoplasms	0.0	0.0	0.0	0.7	0.0	0.5	0.2	0.2	0.2
02.05 and 02.06 Reproductive neoplasm	0.0	0.0	0.0	0.5	0.5	0.5	2.8	2.2	2.6
02.99 Other and unspecified neoplasms	0.2	1.0	0.6	1.6	1.7	1.6	0.5	0.8	0.6
3-Non-communicable diseases	5.1	3.9	4.5	19.8	16.5	18.5	43.1	39.7	41.7
3.01 and 3.02 Severe Anemia and Malnutrition	1.2	0.9	1.1	0.0	0.0	0.0	0.4	0.2	0.3
03.03 Diabetes mellitus	0.4	0.2	0.3	0.6	0.5	0.6	2.3	2.3	2.3
04.01 Acute cardiac disease	0.0	0.0	0.0	4.0	7.0	5.2	6.3	4.5	5.6
04.02 Stroke	0.0	0.0	0.0	2.9	1.4	2.3	10.8	11.3	11.0
5.01 and 5.02 COPD and Asthma	0.0	0.0	0.0	1.5	0.3	1.1	10.4	10.6	10.5
06.01 Acute abdomen	2.0	0.8	1.4	3.9	4.1	4.0	3.8	4.3	4.0
06.02 Liver cirrhosis	0.0	0.3	0.1	1.6	0.9	1.3	0.9	0.6	0.8
07.01 Renal failure	0.0	0.4	0.2	1.9	0.5	1.3	2.4	1.0	1.8
08.01 Epilepsy	1.1	1.1	1.1	1.7	0.6	1.3	0.4	0.2	0.3
Others and unspecified NCDs	0.5	0.3	0.4	1.9	1.1	1.6	5.4	4.7	5.1
4- Maternal and neonatal	37.5	32.9	35.3	0.0	2.3	0.9	0.0	0.0	0.0
09.04 Obstetric hemorrhage	0.0	0.0	0.0	0.0	1.4	0.6	0.0	0.0	0.0
Other and unspecified maternal COD	0.0	0.0	0.0	0.0	0.9	0.3	0.0	0.0	0.0
10.01 Prematurity	14.9	10.8	13.0	0.0	0.0	0.0	0.0	0.0	0.0
10.02 Birth asphyxia	5.7	5.1	5.4	0.0	0.0	0.0	0.0	0.0	0.0
10.03 Neonatal pneumonia	7.9	6.4	7.2	0.0	0.0	0.0	0.0	0.0	0.0
10.04 Neonatal sepsis	2.9	4.2	3.5	0.0	0.0	0.0	0.0	0.0	0.0
10.06 Congenital malformation	1.6	1.4	1.5	0.0	0.0	0.0	0.0	0.0	0.0
10.99 Other and unspecified neonatal	4.5	5.1	4.8	0.0	0.0	0.0	0.0	0.0	0.0
5-Trauma	6.7	7.0	6.8	34.8	41.2	37.3	5.1	4.5	4.9
RTAs and other transport Accidents^a^	1.4	0.8	1.1	13.5	13.1	13.3	1.8	1.7	1.7
12.03 Accidental fall	2.5	1.6	2.1	2.5	1.3	2.0	1.6	1.4	1.5
12.04 Accident drowning and submersion	1.1	1.7	1.4	1.4	3.9	2.4	0.0	0.2	0.1
12.05 Accidental exposure to smoke fire	0.3	0.9	0.6	0.7	3.0	1.6	0.0	0.2	0.1
12.08 Intentional self-harm	0.7	0.0	0.4	11.6	13.6	12.4	0.9	0.5	0.7
12.09 Assault	0.0	0.0	0.0	2.7	4.6	3.4	0.4	0.0	0.2
12.10 Exposure to force of nature	0.0	0.4	0.2	0.4	0.6	0.5	0.0	0.0	0.0
Other and unspecified external COD	0.6	1.4	1.0	2.0	1.1	1.7	0.5	0.5	0.5
6-Indeterminate	13.9	16.2	15.0	20.5	15.1	18.4	20.2	19.3	19.8

a Road traffic accident.

## Discussion

The findings of the present study suggest a declining CSMR of communicable diseases, and an increasing CSMR of non-communicable diseases. Further, non-communicable diseases were the most common COD in this population. This trend was in agreement with various other studies that revealed the transition of the burden of disease from communicable to non-communicable diseases in India (20–22). The Global Burden of Disease 2010 estimates suggest that there is a shift from communicable diseases toward non-communicable diseases in adults in India (23).

ARI and diarrhea continued to be the most common causes of deaths among children (0 to 4 years). Prevailing cultural practices and absence of routine pneumococcal and rotavirus vaccination might be contributing to high mortality due to ARI and diarrhea (24).

Deaths in an economically productive age group adversely affect households as well as society as a whole and therefore prevention of premature deaths should be prioritized (25). In the present study, the commonest COD among adults was trauma, which accounted for one third of all adult deaths. One third of the trauma cases among adults were due to intentional self-harm, and another one third was due to transport accidents. In adult population, intentional self-harm was more common among females (13.6% of all adult female deaths) as compared to males (11.6% of all adult male deaths). Globally, it has been reported that attempted intentional self-harm is commoner in women but intentional self-harm causing mortality is more common in men (26). Ballabgarh HDSS (Salve et al.) reported that the major cause of intentional self-harm was marital conflicts followed by financial constraint, parental conflict, and educational failure (27).

We had assigned COD by a computerized probabilistic model, InterVA-4. Level of agreement of this method with what is considered to be a gold standard for assigning COD using VA (medical death certificate) has ranged from 40 to 83% in different settings. The level of agreement was found to be better when the diseases were classified under broader groups rather than when compared to more specific CODs and also agreement varied for different groups (13, 19, 28, 29). Since we had used a broader classification of diseases, we expect a high degree of agreement with the physician CODs. Present classification also allowed a comparison with the cause- and age-specific mortality rates from other developing countries, especially from other INDEPTH-associated HDSS sites. In our setting, there was a probability of inter-observer bias as the COD was assigned by a number of physicians. With the use of InterVA-4, more consistency and standardization was expected in assigning of COD.

There were certain limitations attached to the study. The proportion of ‘indeterminate’ as a COD was high in the present study, accounting for about one fifth of the total COD. This could be due to the fact that data extracted from the Ballabgarh VA tool was less when compared to the WHO 2012 VA tool, for which InterVA-4 is actually programmed. Second, we do not know whether the use of InterVA-4 was appropriate, since it has not been validated for Indian settings so far. A period of 4 years is very small to document any trend and hence the trend observed in the present study must be very cautiously interpreted. Cause of death may vary in different geographical locations as there are many factors (environmental, social, cultural, etc.) which are restricted to certain geographical location. This might even affect the probability of the IntervA-4 model. Geographical pattern of cause-specific mortality risk was not done in the present study, but it is a potential subject for future research. However, from the previous studies conducted in Ballabgarh HDSS, it is evident that geographical risk is homogenous at Ballabgarh HDSS (25).

## Conclusions

A declining trend in communicable diseases and a rising trend in non-communicable diseases were observed at Ballabgarh HDSS. Most common COD among children, adults, and the elderly were communicable diseases, trauma, and non-communicable diseases. Policy-makers ought to focus on prevention of premature CODs, especially prevention of infectious diseases in children, and intentional self-harm and road traffic accidents in the adult population.
